# Multiscale Quantitative Rheological Analysis of Composition−Temperature Relationships in Borate-Guar Hydrogels

**DOI:** 10.1021/acsapm.5c02807

**Published:** 2025-12-03

**Authors:** María J. Martín-Alfonso, Francisco J. Martínez-Boza, Paul F. Luckham

**Affiliations:** a Pro2TecS-Chemical Process and Product Technology Research Centre, Department of Chemical Engineering, ETSI, Campus de “El Carmen”, 16743 Universidad de Huelva, Huelva 21071, Spain; b Department of Chemical Engineering and Chemical Technology. 4615 Imperial College London. London SW7 2AZ, United Kingdom

**Keywords:** borate-guar, high temperature, activation energy, rheology, hydrogel, cross-linking

## Abstract

Borate-cross-linked guar gum gels exhibit complex viscoelasticity driven by reversible covalent interactions between borate ions and cis-diol groups. Despite their widespread industrial use, limited knowledge of their thermorheological behavior makes it difficult to predict their performance at high temperatures. Here, 0.50 wt % guar gum dispersions with borax-to-guar ratios ranging from 1:1 to 1:8 (i.e., 0.5000−0.0625 wt % borax) were characterized from 25 to 140 °C using oscillatory rheometry. At 25 °C, gels displayed predominantly elastic behavior (*G*′ ≫ *G*″); above 120 °C, viscous behavior dominated due to thermally induced cross-link dissociation. Time−temperature superposition was valid up to 120 °C, and Arrhenius analysis yielded activation energies of 72−85 kJ mol^−1^ for junction relaxation and −10 to −21 kJ mol^−1^ for modulus decay. A borax threshold near 0.1250 wt % delineated weak from strong gel regimes. Steady shear measurements revealed a three-region flow curve, including shear-thickening and shear-thinning regions that depended on temperature and cross-link density. All formulations deviated from the Cox−Merz rule at high shear. These findings support predictive design of thermoresilient borate-guar gels for energy and high-temperature applications.

## Graphical abstract

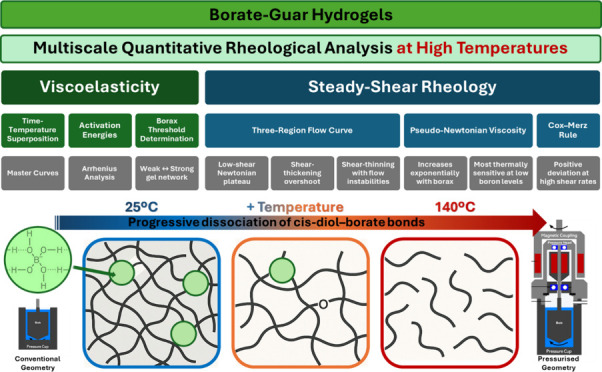

## 1 Introduction

Growing concerns regarding material sustainability have redirected research toward the use of biopolymers. Among them, naturally abundant and biodegradable polysaccharides such as guar gum (GG) are receiving renewed attention for their functional versatility and environmental friendliness.^1,2^ Recent studies have evaluated the life cycle impacts of guar-based formulations across diverse application areas^3,4^

GG is a galactomannan extracted from the seeds of Cyamopsis tetragonoloba, known for its high-water solubility, intrinsic biodegradability, and remarkable thickening capabilities.^5,6^ Structurally, GG consists of a linear backbone of β-D-mannopyranose units linked by (1→4) glycosidic bonds, with α-D-galactopyranose side chains attached via (1→6) linkages.^7^ This architecture enables GG to readily form reversible, viscoelastic networks—properties that make it suitable for applications such as controlled drug release, texture modification, and proppant transport.^8–11^

The rheological behavior of GG solutions is highly dependent on polymer concentration, the presence and density of cross-linkers, and environmental conditions such as temperature and pH.^12–14^ At moderate temperatures, GG solutions typically exhibit pseudoplastic (shear-thinning) behavior, with viscosity decreasing as shear rate increases.^15,16^ As temperature rises, these fluids may approach Newtonian behavior. The introduction of borate and other cross-linkers significantly alters the rheology of GG, yielding hydrogels with tunable viscoelasticity that show G′ values higher than G″ and a flat evolution of G′ with frequency, yet are able to flow owing to their dynamically cross-linked structure. Such rheological modifications are crucial for application-specific designs, particularly in processes that require efficient transport and retention of solids or liquids.^17–19^

Borate ions act as dynamic cross-linkers by forming reversible complexes with vicinal diols on the GG backbone, assembling a three-dimensional network^20,21^ that markedly enhances gel viscosity and elasticity.^17,22^ Increasing borate concentration further augments these mechanical and rheological properties, enabling GG formulations to meet the demands of high-performance environments.^17^ In parallel, elevating the GG content increases the density of diol sites, which in turn enhances the cross-link density when borate is available, contributing to greater resistance to deformation under shear.^23^ Consequently, concentrated formulations are indispensable wherever complex-fluid stability is required during processing or delivery.^24,25^

Temperature plays an important role in the rheology of GG hydrogels because the network structure is very sensitive to high temperature. The ability to manipulate predictively the viscosity and elasticity of borate cross-linked gels as a function of temperature is crucial for applications such as oil extraction and drug-delivery.^10^ In hydraulic fracturing, in particular, an accurate understanding of temperature-dependent fluid dynamics within geological formations is essential in formations where operational temperatures can reach upward of 140 °C, making the rheological stability of guar-gum-based fluids paramount.^18,26^ These fluids must maintain viscosity (100 cP at 100 s^−1^),^27^ effectively suspend proppants, and enable efficient fracturing.^28^ The reversibility of the cross-linked GG network additionally permits the fluid to revert to a low viscosity, simplifying post-treatment recovery.^29^

Recent strategies aimed at improving thermal resilience include the use of heteroatom cross-linkers that outperform traditional borate systems under high-temperature conditions.^18,24^ In addition, introducing hydrophobic moieties into the biopolymer chain markedly alters its thermal and viscoelastic properties, confer greater mechanical stability at elevated temperatures.^27,30^

Hence, understanding how the viscosity and elasticity of such polysaccharide-based hydrogels evolve with temperature represents a fundamental area of research for suitable product design.^29,31^ Dynamic rheological measurements provide valuable insight into how borate-guar gels respond to thermal fluctuations. In general, at moderate temperatures, the storage modulus (*G*′) tends to be higher than the loss modulus (G″), suggesting predominantly elastic characteristics within the gel structure. At elevated temperatures, however, a significant decline in G′ reflects a transition toward viscous-dominated behavior, indicating network degradation and suggesting that the gel integrity is compromised under thermal stress.^32^ The kinetic energy of the molecules increases, accelerating molecular mobility and weakening intermolecular associations.^33^ Additionally, the availability of borate ions decreases at high temperature due to the displacement of the equilibria between boric acid and borate,^20,34–37^ and consequently, the cross-linking density. This leads to the progressive disruption of the network structure with the subsequent diminution in mechanical strength, as evidenced by changes in the rheological profiles of borate-activated gels.^38,39^

Despite recent advances, the role of borate complexation in the solid−liquid transition of guar gum gels at elevated temperatures remains insufficiently resolved, hampering predictive product design based on rheological performance requirement. To redress this knowledge gap, in a previous paper,^16^ the processing and rheological properties of borate-guar gels were determined as a function of temperature and guar/borate ratios. In the present work, we investigate the effect of borate concentration on the rheology of borate-guar gels (BG) at elevated temperatures. Specifically, we: (i) quantify the temperature-dependent kinetics of borate−GG complexation, (ii) correlate molecular kinetics with macroscopic rheological behavior, (iii) estimate activation energies for bond relaxation and cross-link dissociation, and (iv) assess targeted chemical modifications to enhance thermal stability and biostability. This multiscale study investigates the viscoelastic behavior of borate−guar hydrogels over a broad range of time scales and temperatures. By applying the principle of time−temperature superposition (TTSP), rheological data obtained at different temperatures are shifted to construct master curves spanning several decades of frequency. This approach effectively extends the analysis to longer and shorter characteristic times, capturing the material’s dynamics across multiple temporal regimes. The multiscale character of the method lies in its ability to probe thermally activated relaxation mechanisms within the network over varying time and energy scales.

Although GG’s inherent limitations (such as, poor thermal resilience and microbial susceptibility) continue to restrict its industrial utility, these shortcomings can be mitigated through tailored chemical modification strategies.

## 2 Experimental Section

### 2.1 Materials and Sample Preparation

A stock solution of GG at 1 wt % was prepared using a commercial GG supplied by Sigma-Aldrich with *M*_w_ = 2 × 10^6^ g/mol. Sodium tetraborate decahydrate (borax; Sigma-Aldrich) was employed as the cross-linking agent, and sodium azide (0.05 wt %) was added to the solution as a preservative.

The preparation was carried out at ambient temperature in two stages. First, the GG powder was dispersed in deionized water with a Silverson L5 laboratory mixer equipped with a disintegrator head (2000 rpm, 5 min) and then left to hydrate fully for 24 h. Second, the hydrated dispersion was homogenized at 5000 rpm for a further 5 min.

Cross-linked borate-GG gels (BG) were formulated at a GG concentration of 0.5000 wt % (3.08 × 10^−2^ mol/L, based on a sugar unit molecular weight (*Pm*) of 162 g/mol),^37^ with borax concentrations ranging from 0.5000 to 0.0625 wt %. These correspond to borax:guar gum mass ratios of 1:1, 1:2, 1:4, and 1:8, which are equivalent to borate ion (B­(OH)_4_^−^) to sugar unit molar ratios of 3.22, 1.61, 0.80, and 0.40, respectively.

Each gel was prepared by combining the appropriate volume of GG mother solution with deionized water and a 2 wt % borax decahydrate solution, and mixing with a four-blade impeller at 250 rpm and 60 °C for 10 min. Following mixing, the pH was measured and, where necessary, adjusted to 9 by the addition of 0.5 M NaOH. The samples were then cooled to ambient temperature to obtain homogeneous gels, after which the pH was rechecked and readjusted to 9 if required. Samples were stored at 4 °C until use in rheological testing.

### 2.2 Rheological Measurements

Rheological characterization was performed using a Physica MCR-301 controlled stress rheometer (Anton Paar, Austria) equipped with conventional coaxial cylinder geometries (CC27, CC27PR) and pressurized geometries (DG35PR, CC30PR). Conventional coaxial cylinders were used at temperatures of 25, 40, 60, and 80 °C. A solvent trap was employed to minimize evaporative water loss. A pressure cell D400 was used at temperatures between 80 and 140 °C with pressurized geometries. It was pressurized with inert nitrogen at 50 bar to prevent water evaporation at temperatures above 100 °C. The effect of the pressurizing gas was considered negligible by comparing rheological tests for each sample with and without pressurization, using measurements from conventional geometries at 80 °C (Figure S1, Supporting Information).

Frequency sweep tests within the linear viscoelasticity region, previously identified by stress-sweep tests at 1 rad/s, were performed in the range of 0.01−100 rad/s. Steady-state viscosity curves were obtained in both controlled-stress (CS) and controlled-rate (CR) modes by 15 stepwise increases in stress (from 5 μNm to 6·10^4^ μNm for conventional geometries and from 25 μNm to 6·10^3^ μNm for pressure cell) or shear rate (from 10^−3^ s^−1^ to 10^3^ s^−1^), each step lasting 180 s. Samples were introduced into the rheometer at ambient temperature and pressure, then equilibrated for 1 h at the measurement temperature/pressure. Each test was performed in duplicate, with a standard deviation of ± 5% or less among the replicates.

## 3 Results and Discussion

### 3.1 Viscoelasticity

It has been pointed out^27^ that viscosity alone is insufficient to assess the suspension capability of BG gels. In this context, the elastic modulus, which reflects the structural strength of the gel, shows a stronger correlation with suspension performance. Gels with higher elastic moduli exhibit more robust polymer networks and deliver improved sand-transport performance in fracturing operations. Figures 1A and 1B show the evolution of both storage (*G*′) and loss (*G*″) moduli versus frequency, over the temperature range 20−120 °C, for gels prepared at both 0.5000 wt % GG and borax (GG-to-borate mass ratio of 1:1, BG 1:1). At lower temperature (25 °C), the gel exhibits G′ values markedly higher than G″, owing to the large number of borate cross-links that enhance its elasticity (Figure 1A). At the highest temperature examined (120 °C), the storage modulus G′ is markedly lower than the loss modulus G″, signaling that viscous behavior predominates as the number of cross-links diminishes with increasing temperature. This trend reflects the positive activation energy of the borate-association reactions, as earlier noted by Pezron.^40^ The loss modulus G″ also declines with temperature, indicating that the gel becomes less viscous at elevated temperatures owing both to the reduction in borate cross-links and to the greater thermal motion of the polymer chains (Figure 1B).

As the temperature increases, the system undergoes a clear transition from solid to more fluid behavior. Accordingly, the *G*′−*G*″ crossover point shifts to higher frequencies, signifying a loss of gel strength, reduction in elasticity, and faster relaxation caused by weaker entanglements, physical interactions that restrict the motion of individual chains. This response is a key practical feature of BG gels. Above the critical overlap concentration of the GG (c* ∼ 0.5−1.5 g/L),^20,41,42^ increasing the borax content raises both G′ and G″, confirming the formation of a stronger, more interconnected gel network. Two characteristics dominate the resulting frequency sweeps: the weak frequency dependence of G′ and the appearance of a maximum and a minimum in G″. The cross-linking mechanism in GG-B gels is governed by ionic associations between the borate complex and cis-hydroxyl groups along the GG polymer chain, rather than by permanent covalent bonds.^37^ The labile nature of these linkages leads to transient, reversible networks with self-healing capability, owing to the dynamic interaction between borax and the cis-diols of the polysaccharide.^35,40,43,44^ The maximum observed in *G*″, which is an estimate of to the longest relaxation time, reflects the period required for the reversible cross-links to form and dissociate, thereby underpinning the gel’s dynamic character. Reported lifetimes for these associations range from millisecond^21,32,40,45^ to several minutes^46^ allowing the gel to exhibit pronounced self-healing properties.

**Figure 1:**
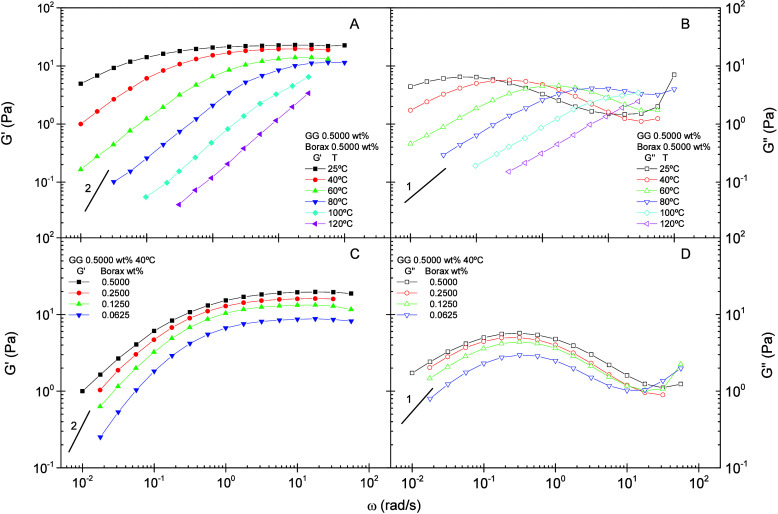
Dynamic storage (*G*′) and loss (*G*″) moduli as functions of frequency, temperature, and borax concentration for borate-guar gels prepared with 0.5000 wt % GG. (A) Storage modulus (*G*′) vs temperature. (B) Loss modulus (*G*″) vs temperature. (C) Storage modulus (*G*′) vs borax concentration. (D) Loss modulus (*G*″) vs borax concentration. The lines are included to guide the eye. Lines 1 and 2 represent slopes in log−log scale.

Figures 1C and 1D depict the variation of the dynamic moduli as functions of frequency for BG gels formulated with different borax contents. Lower borax levels reduce the stiffness of the system, as evidenced by decreases in both moduli and by the narrowing of the frequency interval between the maximum and minimum in G″ across the entire frequency range examined.^10^ These effects arise from a reduced density of cross-links, which yields a less rigid network and diminishes the system’s elastic character. At low borate concentrations, mainly 1:1 complexes are formed, which do not markedly enhance the rheology.^20,21,35,36,47^ Higher borate concentrations promote the formation of 2:1 complexes, leading to effective cross-linking and concomitant increases in gel viscosity and elasticity. It has been noted that a minimum boron content is required to develop gels suitable for fracturing-field uses^27^ and for drug-delivery systems.^8,10^

Conversely, it has been reported that increasing the temperature reduces the boron concentration available for the formation of BG complexes, due to the positive activation energy of the GG-boron equilibrium.^20,48^ Consequently, at a fixed GG concentration gel strength is a function of both temperature and borax content.^25,49^ To go further into the evolution of moduli with borax content across a broad temperature window, Figure 2 displays the storage modulus G′, measured at 10 rad s^−1^ (well within the plateau region), over the full temperature range investigated.

**Figure 2:**
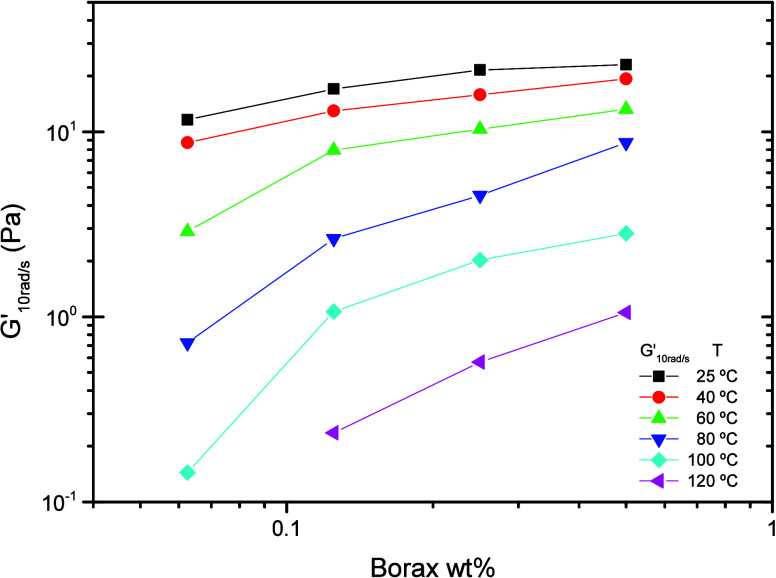
Storage modulus (*G*′), at 10 rad/s, as a function of borax content for BG gels formulated with 0.5000 wt % GG, over the temperature range 25−120 °C. The lines are included to guide the eye.

As can be observed in Figure 2, at 25 and 40 °C, the storage modulus G′ rises slightly with increasing borax content, in agreement with previous work showing that gel strength and network stability are proportional to boron concentration.^21,50^ Although the overall increase is gradual, two distinct linear regimes can be identified in the G′-versus-borax plot: one for borax levels below 0.1250 wt % and another above this threshold, the change in slope being most evident at 60 °C. At temperatures above 60 °C, the divergence between these two regimes becomes more pronounced, indicating a substantial difference in gel strength. Thus, greater borax contents confer higher thermal resistance on the gel. A borax concentration of 0.1250 wt % (equivalent to 268 ppm of boron) appears to be the minimum required for BG gels formulated with 0.5000 wt % GG to meet practical oil-field specifications, in line with previous studies,^27,51,52^ although it is markedly higher than the borax level needed for gelation in the borate-guar system as determined by the Winter−Chambon criterion.^53^

Materials exhibiting thermorheological simplicity permit the interchange of time and temperature for all linear-viscoelastic functions. Consequently, master curves can be constructed at a chosen reference temperature by horizontally shifting, along the frequency axis, the curves of the same linear-viscoelastic function obtained at different temperatures.^54^ The viscoelastic properties of borate-guar (BG) gels conform to both the time−temperature and time−pH superposition principles,^40,48^ thereby requiring the use of horizontal, a_T_, and vertical, b_T_, shift factors. From the practical point of view, master curves constructed with these factors characterize the viscoelastic behavior over broad frequency and temperature windows. This is illustrated in Figures 3A-3B and 3C-3D, where the storage and loss moduli obtained across the complete temperature range collapse onto single curves for borax concentrations of 0.5000 and 0.1250 wt %, respectively. The time−temperature superposition principle thus extends the frequency domain to values that are experimentally inaccessible at the arbitrarily chosen reference temperature.^54^

Master curves obtained for these borate-guar (BG) gels at the reference temperature of 40 °C exhibit both the plateau and terminal regions. Owing to the transient character of the cis-diol−borate cross-links, BG gels form dynamic physical networks; consequently, the system displays a terminal-flow region at frequencies below the inverse of the terminal relaxation time^48^ with slopes of G′ and G″ higher than those predicted by the Maxwell model for the terminal region (G′ ≈ 2 and G″ ≈ 1, in log scale). Some scatter is apparent in the master curves, occurring in the terminal region for gels with higher borax content (Figures 3A and 3B) and in the plateau region for gels with lower borate content (Figures 3C and 3D). This behavior is probably attributable to the presence of several phases with differing cross-link densities, which give rise to relaxation processes displaying only weak time−temperature dependence, thereby deviating the system from strict thermorheological simplicity. Moreover, an experimental artifact associated with instrument inertia is likely responsible for the apparent overdecrease in G′ and overincrease in G″ at higher frequencies. Nevertheless, the superposition may still be regarded as valid, within experimental error, for engineering purposes.

**Figure 3:**
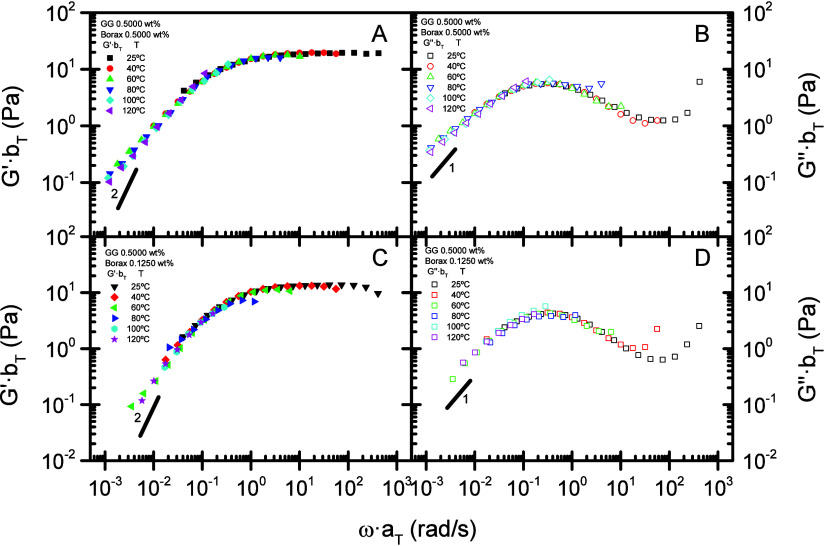
Master curves at the reference temperature of 40 °C, for a BG gel formulated with 0.5000 wt % GG and different borax contents: 0.5000 wt % borax (A, B); 0.1250 wt %, (C, D). (A, C) Storage modulus (*G*′). (B, D) Loss modulus (*G*″). Lines 1 and 2 represent slopes in log−log scale.

The temperature dependence of the horizontal, a_T_, and vertical, b_T_, shift factors was modeled individually using Arrhenius-type equations:

$$\ln a_{T}=\exp\left[-\frac{E_{aa}}{R}\left(\frac{1}{T}-\frac{1}{T_{0}}\right)\right]$$ 1

$$\ln b_{T}=\exp\left[\frac{E_{ab}}{R}\left(\frac{1}{T}-\frac{1}{T_{0}}\right)\right]$$ 2

where *E*_aa_, *E*_ab_ are the activation energies, R is the universal gas constant, and T_0_ is the reference temperature, arbitrarily fixed at 60 °C. *E*_aa_ represents the activation energy for the elementary viscoelastic relaxation process. *E*_ab_ is associated with changes in the plateau modulus caused by variations in the number of cross-linking sites, governed by the borate-diol equilibrium.^55^

Figure 4 illustrates the evolution of both horizontal and vertical the shift factors with temperature, and the corresponding activation energies are listed in Table 1. As has been previously reported by other researchers,^39,48,56–59^ the temperature dependence of the shift factor for BG follows an Arrhenius equation. In the present study a satisfactory fit is obtained for borax contents of 0.1250 wt % and above, whereas poorer fits are observed at lower contents, suggesting that the network formed at low boron concentrations is less structured and therefore more sensitive to changes in shear−temperature. The activation energy, *E*_aa_, derived from the horizontal shift factors increases slightly as the borax content decreases, indicating greater thermal susceptibility in this direction. The *E*_aa_ values are of the same order as those reported by Dawson^37^ and Kesavan^48^ for HPG borate gels and by Hu^58^ for a carboxymethyl hydroxypropyl guar (CMHPG) gel of slightly lower polymer concentration (0.36 wt %), although marginally lower values are obtained in the present work.

**Table 1:** Activation Energies for the Horizontal (*E*_aa_) and Vertical (*E*_ab_) Shift Factors as a Function of Borax Content

borax content (wt %)	BG mass ratio	BG molar ratio	*E*_aa_ (kJ/mol)	*E*_ab_ (kJ/mol)
0.5000	1:1	3.22	71.8	−10.4
0.2500	1:2	1.61	72.6	−15.0
0.1250	1:4	0.80	73.0	−17.9
0.0625	1:8	0.40	85.3	−21.0

The activation energies, *E*_ab_, derived from the vertical shift factors for these BG gels are slightly lower than those reported by Kesavan and Prud’homme^48^ and Pezron and Leibler;^40^ nevertheless, the values do not differ significantly.

**Figure 4:**
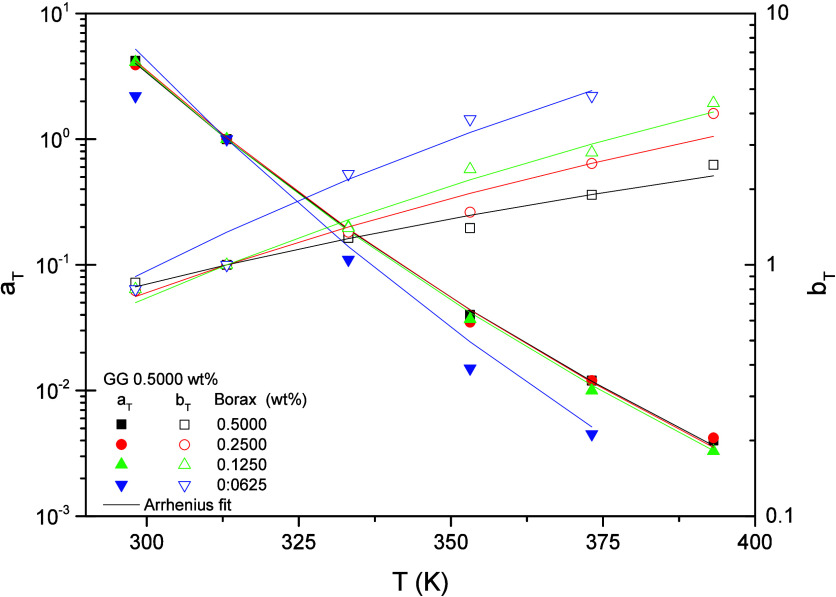
Horizontal (a_T_) and vertical (b_T_) shift factors for BG gels formulated with 0.5000 wt % GG at different BG ratios.

### 3.2 Flow Behavior

Figures 5A and 5B present flow curves for gels prepared with 0.5000 wt % GG containing 0.5000 and 0.0625 wt % borax, respectively, over the temperature range 25−140 °C. It is well-known that adding borax to GG solutions, above a pH-dependent critical concentration,^41^ results in a viscosity increase of several orders of magnitude compared with that of a GG solution at the same polymer concentration, even at the highest shear rates for these fluids.^16^ Three distinct regions are evident in the flow curves. At low shear rates, a pseudo-Newtonian region, in which the viscosity remains almost constant as shear rate increases, is clearly observed. At intermediate shear rates, the gels exhibit a rise in viscosity to a maximum, corresponding to shear-thickening behavior. At high shear rates, a shear-thinning region characterized by a progressive decrease in viscosity with increasing shear rate is observed. Shear thinning arises from the alignment of polymer chains in the direction of flow under the applied stress: chains that are randomly oriented at low shear rates become aligned parallel to the streamlines as the shear rate increases, thereby reducing resistance to flow and lowering viscosity. This high-shear-rate region appears to be independent of borate concentration. Moreover, the viscosity in this region tends toward a limiting value that corresponds to the maximum degree of polymer alignment in the flow direction. It is worth noting that the pseudoplastic drop for these gels exhibits regions of shear rate in which the slope of the flow curve on a log−log scale takes values lower than −1 (see Figure 5). This phenomenon has been attributed to flow instabilities, primarily due to fracture and Weissenberg effects, that reveal bulk heterogeneity in the flowing system. Considering that the tests were carried out using rough geometries, wall-slip effects are expected to contribute to the instabilities to a lesser extent than fracture.^60^ These flow instabilities are responsible for the pronounced viscosity decrease and the accompanying change in slope observed at the end of the shear-thickening zone, after the maximum viscosity attained at each temperature, and at the onset of the pseudoplastic region. At higher shear rates, applied over longer shearing times, the flow becomes stable, and the slope approaches the typical value of about −1 for shear-thinning fluids on a logarithmic scale.

**Figure 5:**
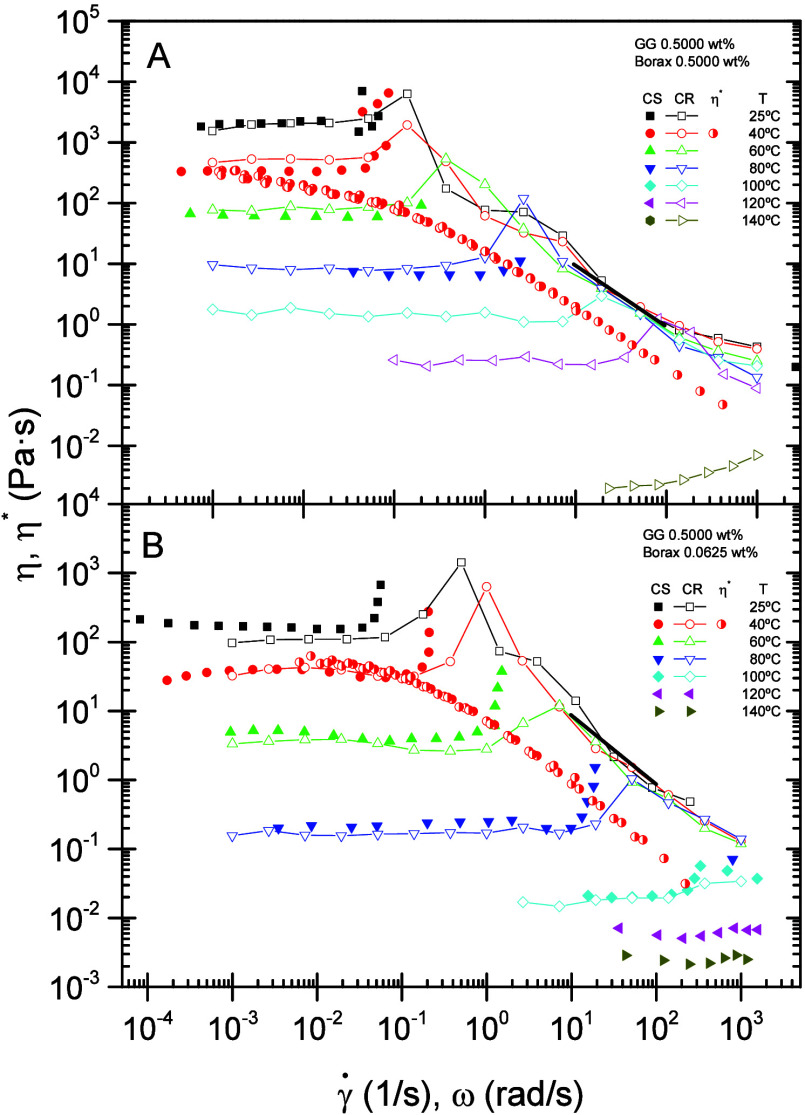
Flow curves as a function of temperature and complex viscosity master curves at 40 °C for BG gels formulated with 0.5000 wt % GG and (A) 0.5000 wt % borax. (B) 0.0625 wt % borax. Solid symbols: viscosity measured in controlled-stress (CS) mode; open symbols: viscosity measured in controlled-rate (CR) mode. The bold black line denotes a slope of −1. The lines are included to guide the eye.

Good agreement between the viscosity values obtained in both controlled-stress and controlled-rate modes is observed, irrespective of borax content and temperature, as shown in Figures 5A and 5B. Tests performed in controlled-stress mode detect the critical shear rate at which the shear-thickening region commences with greater precision. However, this mode is not ideal for characterizing the shear-thinning region because, once a critical stress is exceeded, catastrophic sample fracture occurs, producing a sudden drop in viscosity of several orders of magnitude and yielding spurious, very high apparent shear-rate readings.

As expected, increasing the temperature causes a marked reduction in viscosity. Furthermore, the critical shear rate at which shear thickening occurs shifts to higher values as the temperature rises. Such behavior has also been reported for associative polymers.^56,58,60,61^ However, at higher temperatures the apparent viscosity increase observed for low-viscosity gels is likely an artifact attributable to instrument inertia.^16,28^

The pseudo-Newtonian viscosity of the BG gels studied increases exponentially with boron concentration, as shown in Figure 6, where viscosity values at low shear rate are plotted against borax concentration. Figure 6 also reveals that the thermal susceptibility of the gels, measured as the slope of viscosity versus borax content, is greater at higher temperatures and lower boron concentrations. For the gel formulated with the lowest borax content (0.0625 wt %), the reduction in viscosity (Figure 5B) is less pronounced than the reduction in elastic modulus (Figure 3), indicating that the elastic properties are more sensitive to low cross-link densities when the borax concentration is low.

**Figure 6:**
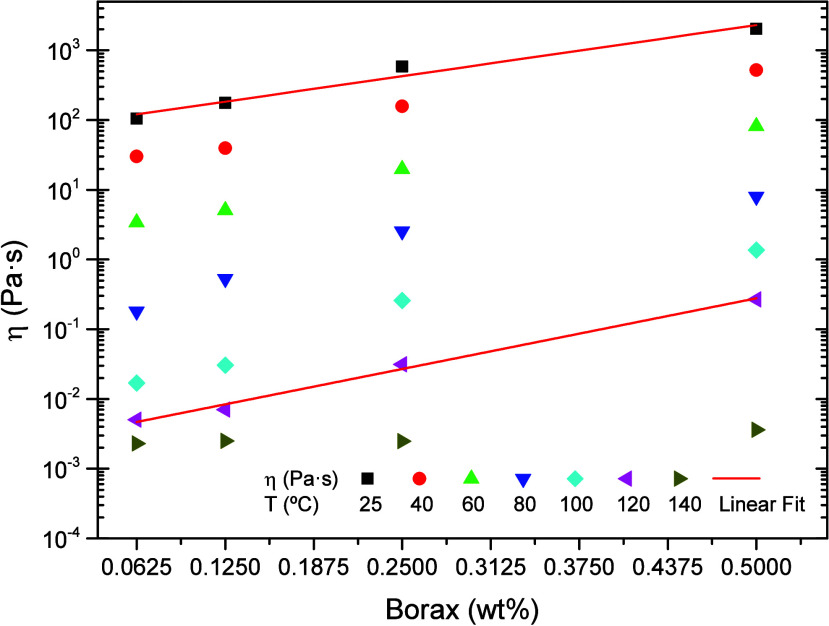
Low-shear-rate viscosity of BG gels at different temperatures.

The transition from the pseudo-Newtonian plateau (constant viscosity at low shear rates) to the shear-thinning region occurs at progressively higher shear rates as the borax concentration decreases, as shown in Figure 7, which plots the critical shear rate at the onset of shear thickening as a function of temperature. At low temperatures, a rise in temperature causes the critical shear rate, $\overset{˙}{\gamma_{c}}$ , to increase almost exponentially, indicating that the gel structure is highly temperature sensitive. This behavior suggests that polymer chains interact more strongly at higher concentrations, forming entanglements that are disrupted under shear.

**Figure 7:**
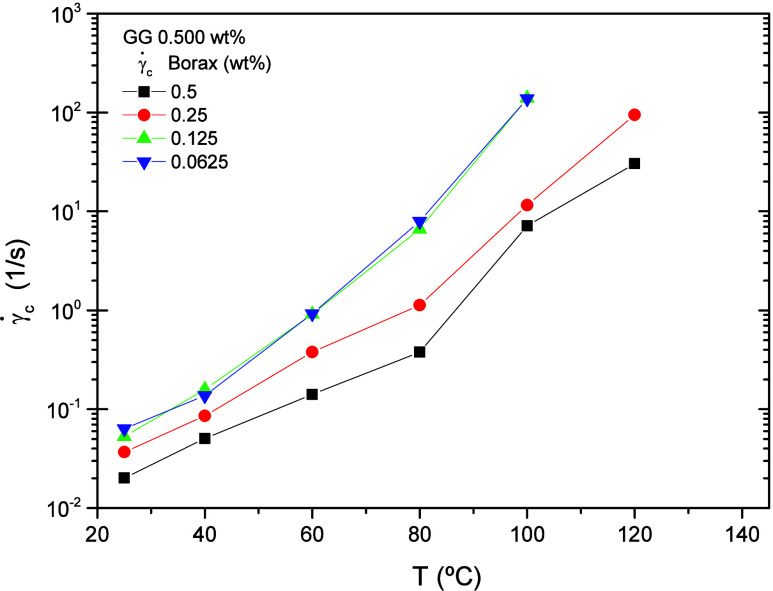
Critical shear rate (onset of shear thickening) of BG gels as a function of temperature. The lines are included to guide the eye.

### 3.3 Structure−Property Relationships

This complex rheological behavior of GG cross-linked by borate ions arises from the dynamic microstructure generated through ion complexation and cross-link bonds between monoborate ions and the cis-hydroxyl groups of GG (2:1 complexes).^20,35,62^ The structure depends on the concentrations of both polymer and borax, as well as on pressure, temperature, and pH, as pointed out by several authors.^13,63–65^ The observed shear thickening has been explained by a shear-induced mechanism in which interchain associations, or cross-link density, increase under flow.^56,58,66,67^ In addition, non-Gaussian chain stretching, whereby GG chains extend beyond the Gaussian regime at high shear rates, contributes to the rise in viscosity and to deviations from the Cox−Merz rule.^68^ After thickening, the subsequent shear thinning may be influenced by flow instabilities,^58^ such as fracture and temporary bulk slip of the fractured phases, which lead to negative flow index values in the power-law region. A schematic of borate−diol cross-linking and its impact on rheological behavior is provided in Figure S2 (Supporting Information).

For a given GG concentration and temperature, the borax content markedly influences shear-flow behavior in GG solutions. High borax concentrations enhance the cross-link density, leading to an increase in baseline viscosity at low shear rates and a decrease in the critical shear rate at which shear thickening begins. Higher boron levels promote stronger interactions that increase viscosity and accelerate network formation, so lower shear rates are required to induce thickening ( $\overset{˙}{\gamma_{c}}$ appears at low shear rate). In addition, increasing the borax concentration raises the amplitude of the shear-thickening peak, a result attributable to the higher cross-link density and the network’s greater resistance to deformation under shear.

For a given BG gel, temperature strongly influences the microstructure and, consequently, the rheological behavior. As the temperature increases, the relaxation time shortens owing to faster molecular motion. This reduction in relaxation time lowers the network’s strength and elasticity and diminishes the extent of shear thickening, because the chains have less time to stretch and form transient associations under shear. Arrhenius relationships derived via Time−Temperature Superposition Principle (TTSP) from oscillatory-shear data predict higher viscosities than those measured in steady flow at temperatures above 100 °C, indicating slight structural degradation over time. Moreover, the decrease in cross-link density and viscosity at elevated temperatures requires higher shear rates to achieve the same level of chain interaction, thus shifting the onset of shear thickening to higher rates and reducing the amplitude of the thickening peak.

BG gels would form dynamic aggregate structures through intermolecular cross-linking. These aggregates behave as flow units with a larger effective volume than individual molecules and therefore exhibit higher viscosity. Under shear, the structures elongate while the cross-linked network is continuously reformed: bonds between adjacent molecules break and reestablish, aligning the structure in the direction of flow. This process generates larger aggregates and higher viscosities without increasing the total number of cross-links at a given temperature and composition.^67^ Shear thickening arises when the time scale of deformation, the inverse of the shear rate, becomes shorter than the relaxation time.

Further increases in shear stress or shear rate sustain the process until the stress reaches a level at which bond rupture outweighs the formation of new linkages. At this point of maximum viscosity, the aggregate size decreases and the viscosity begins its pseudoplastic decline, with a flow index close to zero. In addition, at this stress or shear-rate level, flow instabilities (such as fracture, shear banding and wall slip) may arise, violating the boundary conditions for homogeneous flow and producing a region characterized by negative flow index values. These instabilities occur more frequently in stronger gels at low temperatures and high borax concentrations. Once well within the high-shear-rate or high-stress regime, the aggregate size stabilizes and the viscosity follows typical pseudoplastic behavior, with a flow index close to zero, irrespective of borax content.

These BG gels exhibit a negative deviation from the Cox−Merz rule; that is, the complex viscosity is lower than the steady-shear viscosity at the same angular frequency (or shear rate), as shown in Figures 5A, 5B, and S3 (Supporting Information). This deviation can be attributed to the strong interactions between the different aggregate phases present in the gels. Similar departures have been reported for associative polymers, where flow-induced structural modification promotes intramolecular associative links at high shear rates, with shorter relaxation times polymers^56,67^ and enhanced hydrogen-bonding in carboxymethyl GG and hydroxyethyl GG.^69^ By contrast, the Cox−Merz rule holds for uncross-linked GG^70^ and for carboxymethyl-hydroxypropyl GG solutions at low concentrations but deviates positively at higher concentrations. In such cases, the behavior is explained by the dissolution of a superentangled structure under steady flow.^5^

## 4 Conclusions

This study provides a comprehensive, multiscale characterization of borate-cross-linked guar-gum gels at a fixed polymer loading of 0.5000 wt %, elucidating how borax concentration and temperature jointly determine viscoelastic performance from 25 to 140 °C. Linear oscillatory tests demonstrate that, at 25 °C, raising the borax level from 0.0625 wt % to 0.5000 wt % increases the storage modulus by roughly 3 orders of magnitude and virtually eliminates its frequency dependence, confirming the formation of an extended, load-bearing network. A threshold close to 0.1250 wt % borax (≈ 268 ppm elemental boron) marks the transition between weak and strong regimes; gels above this threshold satisfy the static-modulus criteria commonly imposed on oil-field fluids. Frequency sweeps from four temperatures collapse onto single master curves via the time−temperature-superposition principle, indicating that the system remains thermorheologically simple to at least 120 °C. Horizontal and vertical shift factors obey Arrhenius kinetics, yielding activation energies of 72−85 kJ mol^−1^ for cross-link relaxation and −10 to −21 kJ mol^−1^ for modulus decay; both quantities increase modestly as the borax content is reduced, showing that sparsely cross-linked networks are more susceptible to thermal softening.

Nonlinear flow measurements reveal the canonical triregion rheogram: a low-shear Newtonian plateau, a pronounced shear-thickening overshoot, and a high-shear thinning zone. The pseudo-Newtonian viscosity grows exponentially with borax concentration, whereas its thermal sensitivity is greatest at low boron levels. The critical shear rate at which thickening begins rises exponentially with temperature but falls with cross-link density, reflecting the competing effects of faster bond exchange and increased network connectivity. The shear-thinning branch exhibits slopes steeper than −1 on a log−log plot, behavior traced to flow instabilities (principally, fracture and transient bulk slip) rather than to wall depletion. All formulations deviate negatively from the Cox−Merz rule (η*­(ω) < η­(γ̇) (*with* ω = γ̇), a signature of shear-induced restructuring in transient physical networks. Above 120 °C, viscous behavior dominates even in highly cross-linked samples, foreshadowing the irreversible bond scission observed near 140 °C; nevertheless, the network remains sufficiently robust up to that limit provided the borax concentration is at least 0.5000 wt %.

The quantitative maps of modulus, viscosity and critical shear rate generated here furnish design rules for tailoring guar-borate fluids to specific tasks. In hydraulic-fracturing and drilling applications, 0.1250 wt % borax represents the minimum dosage for reliable proppant or cuttings suspension at 120 °C, whereas 0.5000 wt % extends operational stability to ultradeep wells at the cost of higher pumping friction. In the biomedical arena, the predictable viscoelastic response enables injectable hydrogels whose stiffness may be tuned in situ by temperature or pH, facilitating controlled drug delivery and soft-tissue engineering.

Two intrinsic limitations remain: irreversible bond cleavage above ∼ 140 °C and the microbial vulnerability of native guar. These constraints point to future research on hybrid or heteroatom cross-linkers, hydrophobic or cationic derivatization of the guar backbone, and real-time scattering studies under combined high-pressure/high-temperature conditions. Even so, the present work establishes a robust mechanistic foundation for the predictive formulation of environmentally benign, thermally resilient polysaccharide gels across energy, industrial and biomedical sectors.

## Supplementary Material



## Notes

Comparison of dynamic moduli measured using conventional and pressurized geometries at 80 °C (Figure S1); structure−property relationships of borate-guar gels as a function of shear rate (Figure S2); steady-state flow curves and complex-viscosity master curves at 80 °C as a function of borax concentration (Figure S3); and crossover frequency and moduli values for the hydrogels studied (Table S1) (PDF)
