# Supplementary material for: Multiscale Quantitative Rheological Analysis of Composition−Temperature Relationships in Borate-Guar Hydrogels
Source: ACS Appl Polym Mater. 2025 Dec 3;7(23):15896–905. doi: 10.1021/acsapm.5c02807 (PMC12707247; doi:10.1021/acsapm.5c02807)
Supplement: Supplementary file 1 [file ap5c02807_si_001.pdf]

Supporting Information

Journal: ACS Applied Polymer Materials

Manuscript ID: ap-2025-028075

Date: 30-Oct-2025

Multiscale Quantitative Rheological Analysis of Composition-Temperature Relationships in Borate-Guar Hydrogels

María J. Martín-Alfonso[a]*, Francisco J. Martínez-Boza[a], Paul F. Luckham[b]

[a] Pro^{2}TecS-Chemical Process and Product Technology Research Centre, Department of Chemical Engineering, ETSI, Campus de "El Carmen", Universidad de Huelva, 21071 Huelva, (Spain).

[b] Department of Chemical Engineering and Chemical Technology. Imperial College London. London, SW7 2AZ (United Kingdom).

* Corresponding author, mariajose.martin@diq.uhu.es

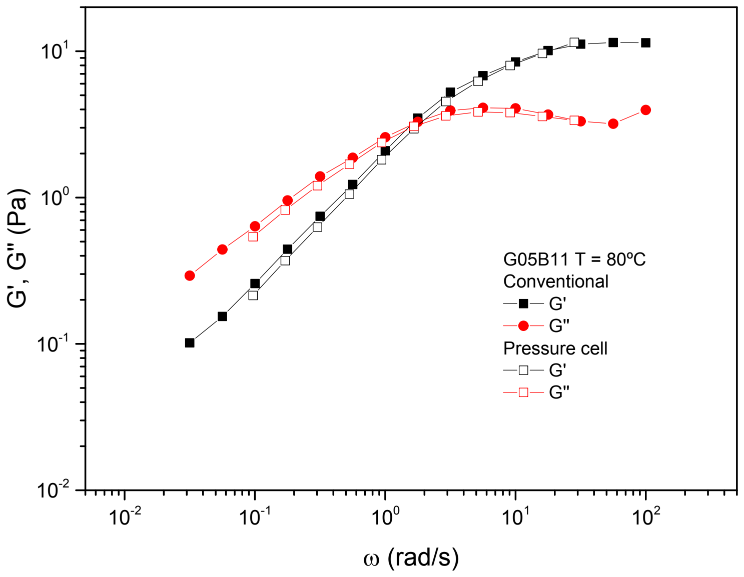

Figure S1. Comparison of the dynamic moduli measured using conventional coaxial cylinder geometry and the pressurised cell at 80°C, for borate-guar gel samples prepared with 0.5000 wt% of both GG and borax.

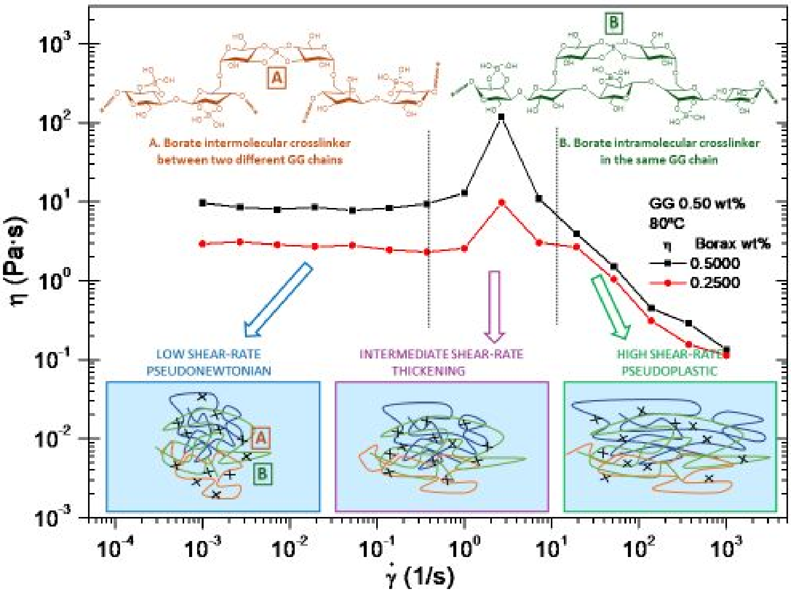

Figure S2. Structure-properties relationships of a BG gel as a function of shear rate.

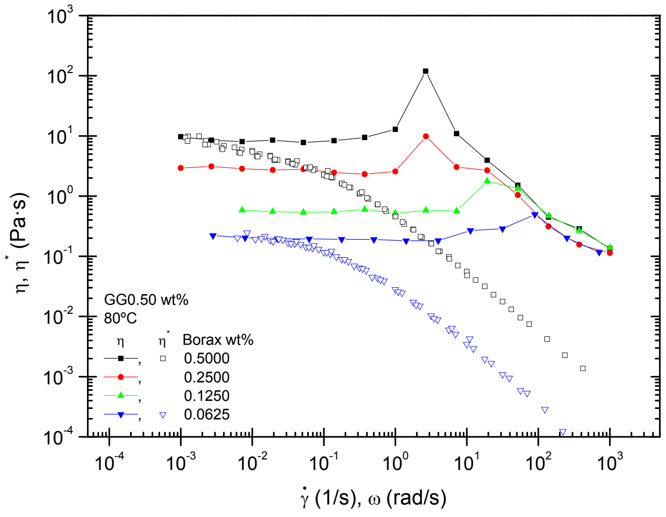

Figure S3. Steady-state flow curves (viscosity versus shear rate) and dynamic viscosity master curves (viscosity versus frequency) at 80°C, plotted as a function of borax concentration.

Table S1. Crossover frequency and moduli values for the hydrogel studied.

	BG 1:1		BG 2:1		BG 4:1		BG 8:1	
T (ºC)	ω (rad/s)	G'=G'' (Pa)	ω (rad/s)	G'=G'' (Pa)	ω (rad/s)	G'=G'' (Pa)	ω (rad/s)	G'=G'' (Pa)
25	--	--	0.02	5.01	0.03	4.42	0.08	3.55
40	0.06	4.30	0.09	4.26	0.12	3.76	0.16	2.61
60	0.36	3.55	0.52	3.35	0.71	2.77	1.42	1.19
80	1.53	3.10	2.59	2.56	3.12	1.40	8.93	0.65
100	5.79	2.37	6.97	1.67	--	--	--	--
120	14.88	1.78	18.77	1.07	--	--	--	--
